# Combining least absolute shrinkage and selection operator (LASSO) and principal-components analysis for detection of gene-gene interactions in genome-wide association studies

**DOI:** 10.1186/1753-6561-3-s7-s62

**Published:** 2009-12-15

**Authors:** Gina M D'Angelo, DC Rao, C Charles Gu

**Affiliations:** 1 Division of Biostatistics, Washington University School of Medicine, 660 South Euclid Avenue, St. Louis, Missouri 63110, USA

## Abstract

Variable selection in genome-wide association studies can be a daunting task and statistically challenging because there are more variables than subjects. We propose an approach that uses principal-component analysis (PCA) and least absolute shrinkage and selection operator (LASSO) to identify gene-gene interaction in genome-wide association studies. A PCA was used to first reduce the dimension of the single-nucleotide polymorphisms (SNPs) within each gene. The interaction of the gene PCA scores were placed into LASSO to determine whether any gene-gene signals exist. We have extended the PCA-LASSO approach using the bootstrap to estimate the standard errors and confidence intervals of the LASSO coefficient estimates. This method was compared to placing the raw SNP values into the LASSO and the logistic model with individual gene-gene interaction. We demonstrated these methods with the Genetic Analysis Workshop 16 rheumatoid arthritis genome-wide association study data and our results identified a few gene-gene signals. Based on our results, the PCA-LASSO method shows promise in identifying gene-gene interactions, and, at this time we suggest using it with other conventional approaches, such as generalized linear models, to narrow down genetic signals.

## Background

The goal of this paper is to develop and evaluate prediction methods and tools for genome-wide association studies, particularly for variable selection and dimension reduction. There is a demand for statistical techniques capable of handling large volumes of data in genetic studies. Technical advances have enabled the collection of massive high-dimensional datasets in such studies. This has called for broadening of the area of research in dimension-reduction techniques to provide methods for prediction and variable selection. For example, during the last decade, Li [1], Tibshirani [2], and Efron et al. [3] have paved new directions for dimension-reduction techniques and broadened the area to other applications of prediction, including genetics.

For this paper, we explore extensions of currently existing dimension-reduction methods and variable-selection methods related to genome-wide association studies (GWAS) single-nucleotide polymorphism (SNP) selection and gene-gene interactions for application to the disease classification problem based on genetic data. Recently, the focus has shifted to GWAS, where the emphasis can be placed on assessing whether multiple markers function together rather than depending on univariate tests and generalized linear models (GLM). Dimension-reduction techniques are a powerful tool because they provide a summary measure of massive amounts of data. We can apply such techniques to determine whether multiple marker pathways and gene-gene interactions are associated with the disease of interest. The highly dense genetic marker data from the rheumatoid arthritis study and the published reports about the study provide an ideal empirical dataset for developing and testing extensions of dimension-reduction methods.

There is a demand for statistical techniques to handle large volumes of data, particularly in the area of genetics. Genetic data is used to find genetic variants that are associated with rheumatoid arthritis risk (or other diseases) through the use of statistical modeling. The tendency for analyzing genotype data is to use GLM and univariate tests; however, these models perform poorly when analyzing high-dimensional data [4,5]. The research objective of this study is to develop prediction tools primarily methods for variable selection and dimension reduction in a GWAS.

In an effort to improve variable selection, Tibshirani [2] developed the least absolute shrinkage and selection operator (LASSO), a penalized likelihood approach, for linear regression. Two important components of variable selection are prediction accuracy and interpretation. Ordinary least squares (OLS) is known to estimate coefficients with small bias but inflated variance. In the case of a large number of predictors, OLS has difficulty selecting the subset of predictors that appears to be the most important or to have the strongest effects. LASSO is a combination of ridge regression and subset selection developed to improve OLS by shrinking the coefficient values and setting some equal to zero. LASSO [2,6] is similar to OLS with constraints and produces a stable and interpretable model. Nonlinear extensions of the LASSO exist such as modeling a binary outcome [6]. Principal-components analysis (PCA) is a nonparametric dimension-reduction approach. PCA is a linear transformation of the original data that incorporates second-order statistics to determine the optimal components that describe the functional relationship between the outcome and covariate [7]. The premise of PCA is to identify the orthogonal linear combinations with the largest covariance. The benefit to using PCA and LASSO is that both methods can accommodate correlation, such as linkage disequilibrium (LD), between SNPs. This advantage prompted us to select PCA and LASSO to model SNPs and genes; models such as GLM fail in the presence of LD [4].

We investigate PCA [7] and LASSO [2,6,8] methods to reduce the dimension of the genetic marker data and detect gene-gene interaction signals on chromosome 6. We explore the two methods, PCA and LASSO, combining variable selection and dimension-reduction techniques. The combined approach will further reduce the dimension of the data to detect signals from variants and gene-gene interactions in addition to the gene(s) discovered in the previously published work on rheumatoid arthritis [9,10]. The bootstrap will be used to estimate the standard errors and confidence intervals of the LASSO coefficient estimates. We will compare the LASSO-PCA approach to the LASSO method including the entire set of SNP values, the logistic regression with individual PCA-PCA interaction, and the logistic regression with individual SNP-SNP interaction.

## Methods

We denote *Y*_*i *_∈ {0, 1} to be the outcome and *Z*_*i*, *k *_= {0, 1, 2}, *k *= 1,...,*K*, to be the SNP variables of a *K*-dimensional covariate vector *Z*_*i *_= (*Z*_*i*,1_,...,*Z*_*i*, *k*_)^*T *^with *n *subjects, where *i *= 1,..,*n *is the subscript for the *i*^th ^subject. Logistic regression is the model of choice for a binary outcome and it is a member of GLM. We specify *Y *to have a binomial distribution, *Y*_*i *_~*bin*(*n*, *μ*_*i*_), where the mean is , the linear predictor is given by , and the link function here is the logit function of the form *g*(*μ*_*i*_) = ln [*μ*_*i*_/(1-*μ*_*i*_)] = *η*_*i*_. The link function describes the relationship between the mean of the distribution function and the linear predictor. The log-likelihood is of the form

LASSO was originally intended for linear regression and it has been extended to the GLM by Lockhorst [6]. The LASSO and GLM algorithms are combined to provide a generalized LASSO algorithm [6] to estimate the LASSO coefficients. The idea is to use an iteratively reweighted least-squares approach to compute estimates of the regression coefficients in a LASSO model while placing a constraint on the regression coefficients. The generalized LASSO algorithm begins with initial estimates of  and , where *h*_*i *_is a specified weight. Initial values of *β *are not needed. Another option it to start with coefficient values of 0; however, this can take too long to converge. The covariates that are not constrained can be swept out. We denote these covariates as *V*_*i*_, and their regression coefficient parameters as denoted *β*. The covariates that are constrained are denoted as *X*_*i*_, and the regression coefficient parameters are denoted *γ*. The next step is to estimate the adjusted response variable, , that is of the form , where *j *denotes the iteration number, *a *denotes adjusted, and . The next step involves projecting the weighted independent variables and weighted adjusted dependent variable onto the column space of , where *W *is a weight and of the form  and . The updated covariates and response variable are given by  and  The regression coefficients for *V *are estimated as *β *= (*V*^*T*^*W*^(*j*)^*V*)^-1^*V*^*T*^*W*^(*j*)^*Y*^*a*,(*j*)^. The last step is to solve min(Y^*,(*j*) ^- X^*,(*j*)*γ*^)^*T*^(Y^*,(*j*) ^- X^*,(*j*)*γ*^) subject to ||*γ*||_1 _≤ *t*. The tuning parameter, *t *> 0, specifies the amount of shrinkage that will be applied to the coefficient estimates. The tuning parameter is estimated by selecting a normalized parameter, *s*, that is the ratio of the tuning parameter to the total effect size of the regression unbounded estimate, which is expressed as *s *= *t*/||(X^*T^X*)^-1 ^X^*T^X*||_1_, 0 ≤ *s *≤ 1. It should be noted when *s *= 1 there is no shrinkage. The estimates are updated and the iterative process is continued until convergence.

For each gene, the score derived from the PCA is a linear combination of the SNPs. This PCA score represents a summary measure of the SNPs from the *g*^th ^gene in a condensed fashion, where the score is  is the *l*^th ^PCA component for the *g*^th ^*gene*, and *Z*^(*g*) ^is the raw SNP data from the *g*^th ^gene. The components that account for at least 10% of the variance are chosen, where *D*_*l*, *g *_is the summary measure to determine the percentage of variance for the *g*^th ^gene, , and *d*_*l*, *g *_denotes the eigenvalues obtained from the PCA for the *g*^th ^gene.

The R package we used for analysis is LASSO2. LASSO2 has limited capabilities when analyzing categorical data, such as the inability to estimate the standard errors. As recommended by Meier et al. [11], we used the bootstrap [12] to estimate the standard errors and confidence intervals. A non-zero LASSO coefficient value indicates the variable should be considered for variable selection and further investigation is necessary. The bootstrap confidence interval can indicate the statistical importance of a covariate from the LASSO. We selected *C *= 1000 bootstrap samples from the data (*Y*, *Z*, *S*) with replacement. For each of these bootstrap samples, we estimated the LASSO coefficient  for the *c*^th ^bootstrap sample where *c *= 1,...,*C *and the star (*) indicates the estimate is from the bootstrap. The average of the bootstrapped estimate is [12]. The variance of the bootstrapped estimate is [12]. An estimate of the bias of *θ *is [12]. The normal-theory interval is used to estimate the 95% bootstrap confidence interval. We assume *θ *has a normal distribution, , and the confidence interval is of the form [12].

## Results

The *HLA-DRB1 *gene on chromosome 6 has been linked to rheumatoid arthritis [9]. Based on this finding, we decided to evaluate markers from chromosome 6. We focused on markers from a subset of the genes that were explored in studies conducted from 1992 to 2003 [9]. A total of 135 SNPs were considered for analysis from 28 genes: *AP *(n = 1), *HLA *class (n = 16), *MICA-MICF *(n = 6), *TAP *(n = 2), and *TNF *(n = 3). PLINK has been used for quality control. From chromosome 6, there are 35,574 markers, with 33,585 SNPs left after removing those that failed the Hardy-Weinberg equilibrium test (*p *≤ 0.001), the missingness test (GENO > 0.1), and the frequency test (minor allele frequency < 0.01). We have removed the SNPs that did not meet the quality control criteria.

The intercept was the only variable swept out in the LASSO model. The number of components selected with PCA ranged from one to two. All PCA scores and the corresponding PCA_*gene*_*a*_-PCA_*gene*_*b *_interactions were entered into the LASSO model to determine whether there was gene-gene interaction. Here, the PCA_*gene*_*a *_is a PCA score from the *a*^th ^gene and the PCA_*gene*_*b *_is a PCA score from the *b*^th ^gene, where *a *≠ *b*. Table 1 has the results indicating 16 potential interactions with their bootstrap standard error and bootstrap confidence interval. Based on the bootstrap estimates, only two gene-gene interactions of *HLA-DRA *× *HLA-DRB9 *and *HLA-DRA *× *MICA *were significant. Of these 16 potential gene-gene interactions, we entered the raw SNP values and the corresponding SNP_*gene*_*a*_-SNP_*gene*_*b *_interactions into the LASSO model to determine whether the same genetic relationships exist. Here, the SNP_*gene*_*a *_is a SNP from the *a*^th ^gene and the SNP_*gene*_*b *_is a SNP from the *b*^th ^gene, where *a *≠ *b*. Eleven gene-gene interactions were suggested from the LASSO-SNP method, while three of these gene-gene interactions were suggested from the LASSO-PCA analysis. However, the final results are the same from both the LASSO-PCA and LASSO-SNP method, where there were two significant gene-gene interactions of *HLA-DRA *× *HLA-DRB9 *and *HLA-DRA × MICA*. We did explore selecting the components using a scree plot; it often selected too many components with noise. In addition, we set the value of the normalized parameter to 0.5 and explored various normalized parameter values to determine the optimal value for variable selection. Our analysis was inconclusive on the best measure to select an optimal value and we will explore this further in the future.

**Table 1:** LASSO results

	LASSO/PCA approach		LASSO/SNP approach	
		
Gene × Gene	Lasso coef (SE)	CI	Lasso coef (SE)	CI
*HLA-B × HLA-DQB1*^a^	-0.0446 (0.0600)	(-0.162, 0.073)		
*HLA-B × HLA-DRA*	0.0452 (0.0781)	(-0.108, 0.198)		
*HLA-DMB × HLA-DQA2*^b^	-0.0044 (0.0435)	(-0.090, 0.081)		
*HLA-DPA1 × MICA*	0.0197 (0.0261)	(-0.031, 0.071)		
*HLA-DPB1 × MICA*^a, b^	0.1047 (0.0601)	(-0.013, 0.223)		
*HLA-DQA2 × TNFRSF21*	-0.0356 (0.0230)	(-0.081, 0.010)		
*HLA-DQB1 × TNF*	-0.1848 (0.1073)	(-0.395, 0.026)		
*HLA-DRA × HLA-DRB9*^a, b^	0.2334 (0.0817)	(0.073, 0.394)^c^	-0.3574 (0.0872)	(-0.528, -0.186)^c^
*HLA-DRA × MICA*^a, b^	-0.1125 (0.0528)	(-0.216, -0.009)^c^	-0.2665 (0.0826)	(-0.428, -0.104)^c^
*HLA-DRA × TNF*	0.1214 (0.0933)	(-0.062, 0.304)		
*HLA-DRA × TNFRSF21*	-0.0252 (0.0232)	(-0.071, 0.020)	-0.0078 (0.0465)	(-0.099, 0.083)
*HLA-F × MICD*	-0.0042 (0.0435)	(-0.089, 0.081)		
*MICA × MICB*^a, b^	-0.0307 (0.0330)	(-0.095, 0.034)		
*MICA × TNF*	0.0280 (0.0514)	(-0.073, 0.129)		
*MICA × TNFAIP3*	-0.0179 (0.0230)	(-0.063, 0.027)		
*TAP2 × TNF*	0.0202 (0.0354)	(-0.049, 0.090)		
*HLA-DPB1 × TAP2*^a^			0.0372 (0.0812)	(-0.122, 0.196)
*HLA-DQB1 × HLA-DRA*^a^			-0.0068 (0.0433)	(-0.092, 0.078)
*HLA-DQB1 × MICD*			-0.0316 (0.0417)	(-0.113, 0.050)
*HLA-DRA × TAP2*^a^			-0.0715 (0.0565)	(-0.182, 0.039)
*MICA × TAP2*^a^			-0.0482 (0.0434)	(-0.133, 0.037)
*MICB × MICD*^a^			0.0478 (0.0539)	(-0.058, 0.153)
*MICB × TAP2*^a^			0.0933 (0.0768)	(-0.057, 0.244)
*MICD × TAP2*			0.1257 (0.0933)	(-0.057, 0.309)

^a^Logistic model with significant SNP_*gene*_*a*_-SNP_*gene*_*b *_interactions

^b^Logistic model with significant PCA_*gene*_*a*_-PCA_*gene*_*b *_interactions

^c^Significant finding; *gene_a *is the *a*^th ^gene and *gene_b *the *b*^th ^gene where *a *≠ *b*

Additionally, we ran logistic regression models with the individual SNP_*gene*_*a*_-SNP__*gene*_*b *_interactions and the individual PCA_*gene*_*a*_-PCA_*gene*_*b *_interactions to compare methods. A multiple-comparison procedure was applied using the Benjamini and Hochberg [13] method, which controls the false-discovery rate. With the individual SNP-SNP interaction from the logit model, we found 337 significant interactions that reduced to 78 unique gene-gene interactions; out of these, 11 overlapped with the LASSO findings. For the individual PCA_*gene*_*a*_-PCA_*gene*_*b *_interactions in the logit model, we found 37 gene-gene interactions and only 5 overlapped from the LASSO findings. The two gene-gene interactions consistently found to be significant across all four approaches were *HLA-DRA *× *HLA-DRB9 *and *HLA-DRA × MICA*. This suggests that the individual SNP-SNP interactions may function jointly instead of independently. Further investigation of the LASSO and PCA approach will be pursued. A third approach was explored that involved placing all 135 SNPs in a LASSO model to determine whether there were any variant-variant signals. There were limitations to this approach due to the large amount of categorical data and large number of interactions. We did not pursue this method much further after recognizing the analysis had to be split into three LASSO models.

## Conclusion

In GWAS there is an overwhelming amount of data and it can be difficult to distinguish between true signals and spurious results based only on single-marker analysis. Our approach is focused on assessing whether multiple markers act together in producing the phenotype. We demonstrate a combined approach of a dimension-reduction method, PCA, and a variable-selection method, LASSO, to detect gene-gene interaction signals. We have extended the LASSO method to estimate standard errors and confidence intervals with the bootstrap.

Interestingly enough, whether the principal-component score or the raw SNP values were placed into the LASSO, the final results were the same. The results from the individual interaction PCA logit models and individual interaction SNP logit models had overlapping results and revealed the same interactions found in the LASSO method. This suggests the PCA-LASSO method shows promise. At this time we suggest using it with other conventional approaches to narrow down genetic signals. The advantage to our method is that highly collinear data and a large number of variables can be reduced to a manageable dimension, where LD is accommodated by LASSO and PCA. Also, a large number of SNPs can be represented as a function of a gene.

A limitation of this current work is that we cannot conclude whether our PCA-LASSO method is an improvement over other gene-gene variable-selection methods. We will further investigate the threshold of the number of covariates in the LASSO model. We propose to do simulation studies in the future that will compare the PCA-LASSO approach to other variable selection methods [4,11]. Simulation studies are necessary to determine the properties of the PCA-LASSO approach. We will also pursue study of the normalized parameter in our future work.

## List of abbreviations used

GLM: Generalized linear models; GWAS: Genome-wide association studies; LASSO: Least absolute shrinkage and selection operator; LD: Linkage disequilibrium; OLS: Ordinary least squares; PCA: Principal-components analysis; SNP: Single-nucleotide polymorphism.

## Competing interests

The authors declare that they have no competing interests.

## Authors' contributions

GMD conceived the study, extended the methodology, performed the statistical analysis, interpreted the results, and drafted the manuscript. DCR participated in revision of the manuscript and interpretation of the results. CCG participated in the study design, interpretation of the results, and revision of the manuscript. All authors read and approved the final manuscript.
